# Heart rate optimization to reduce tricuspid regurgitation in patients with atrial fibrillation and relative bradycardia: A case report

**DOI:** 10.1016/j.jccase.2025.05.011

**Published:** 2025-06-13

**Authors:** Daisuke Nagatomo, Akihito Ishikita, Ryo Miyake, Masatsugu Nozoe, Keiji Oi, Nobuhiro Suematsu, Toru Kubota

**Affiliations:** Department of Cardiology, Saiseikai Fukuoka General Hospital, Fukuoka, Japan

**Keywords:** Tricuspid regurgitation, Atrioventricular valve inflow, Optimal heart rate, Relative bradycardia

## Abstract

A 74-year-old male with end-stage renal disease was referred for treatment of syncope and hypotension episodes during dialysis. The patient had a history of sick sinus syndrome that was managed with a VVI pacemaker, maintaining a heart rate of approximately 60 bpm, due to atrial fibrillation. Transthoracic echocardiography revealed massive tricuspid regurgitation (TR), which was identified as a significant contributor to the patient's symptoms. Surgical intervention for TR was initially considered, however echocardiographic examination with pulse Doppler of the tricuspid inflow waveform indicated that ventricular filling efficiency could be improved by increasing the pacemaker's heart rate to 80 bpm. This adjustment was validated during right heart catheterization, confirming enhanced efficiency and leading to the decision to monitor the patient's condition with the new pacemaker setting instead of proceeding with surgery. Over the next 5 months, the patient's condition significantly improved, with TR severity decreasing to moderate. This case highlights the importance of tailored heart rate optimization in managing complex heart failure, demonstrating the effectiveness of noninvasive methods in improving outcomes for patients with significant tricuspid valve disease and relative bradycardia with atrial fibrillation.

**Learning objective:**

Assessing the optimal heart rate in patients with heart failure is crucial, requiring a case-by-case evaluation rather than relying on evidence from large clinical trials. In this case, characterized by relative bradycardia with chronic atrial fibrillation and severe tricuspid valve regurgitation, we determined the optimal heart rate using the Doppler waveform of the tricuspid valve inflow to assess whether an increase in heart rate could enhance cardiac output without reducing stroke volume.

## Introduction

Severe tricuspid regurgitation (TR) in patients with atrial fibrillation (AF) and relative bradycardia poses a therapeutic challenge, especially when invasive procedures are not preferred. In selected cases, noninvasive strategies such as heart rate optimization may offer effective symptom relief and hemodynamic improvement. Here, we present a case illustrating the utility of Doppler echocardiography-guided heart rate adjustment in managing TR associated with AF.

## Case report

A 74-year-old man with chronic glomerulonephritis on dialysis for 2 years presented with intradialytic hypotension, complicating dry weight achievement and causing pleural effusion. Ten years previously, he was diagnosed with sick sinus syndrome, and a VVI pacemaker was implanted. He recently experienced post-dialysis syncope and was referred to our hospital. Echocardiography revealed massive tricuspid regurgitation (TR), contributing to right heart failure and significant hypotension during fluid removal. TR was attributed to atrial functional TR based on right atrial enlargement without evidence of lead impingement (Fig. 3A, Video 1). A 3D echocardiogram confirmed that the pacemaker lead did not interfere with the tricuspid leaflets, ruling out device-induced TR (Fig. 3G).

**Fig. 1:**
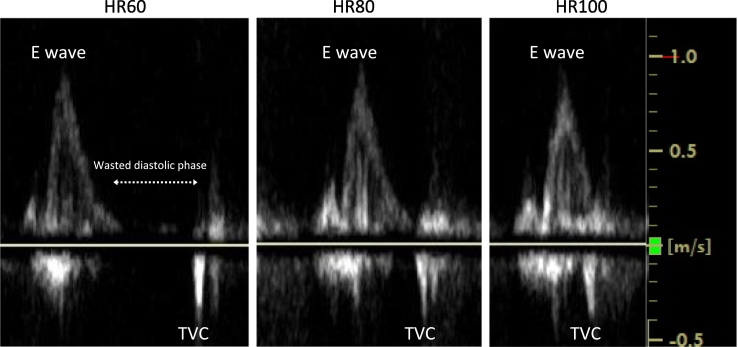
This figure illustrates the Doppler waveform of the tricuspid valve inflow at three heart rates: 60, 80, and 100 bpm. The inefficiency gap between the end of the E wave and the closure of the tricuspid valve is evident at 60 bpm, indicating suboptimal ventricular filling. Adjusting the heart rate to 80 bpm eliminates this gap, improving ventricular filling efficiency. At 100 bpm, further adaptations in waveform indicate the negative effects of increased heart rate on ventricular filling dynamics. HR, heart rate; TVC, tricuspid valve closure.

Echocardiography showed massive TR with leaflet separation and a vena contracta width of 15.2 mm. Right ventricular function was preserved, with a tricuspid annular plane systolic excursion (TAPSE) of 34 mm and a right ventricular fractional area change (RV-FAC) of 49 %. Based on these findings, surgical intervention for symptomatic TR was initially considered. However, Doppler analysis of tricuspid inflow showed a gap between the E wave and valve closure, indicating inefficient diastolic filling (Fig. 1). This inefficiency, along with an AF rate of ∼60 bpm, suggested the need for a higher pacing rate. Increasing the rate to 80 bpm eliminated the gap, aligning valve closure with the end of the E wave and improving filling. At 100 bpm, premature valve closure interrupted the E wave, indicating 100 bpm was too fast (Fig. 1). Thus, 80 bpm was determined to be optimal.

Other options, including defibrillation followed by rhythm control, and physiologic pacing such as His bundle or left bundle branch pacing, were considered. Although biventricular systolic function was preserved, these approaches were evaluated due to anticipated long-term RV pacing dependence. These invasive options were avoided due to the patient's persistent AF, advanced age, and preference to avoid invasive procedures. Therefore, rate control was prioritized.

**Fig. 2:**
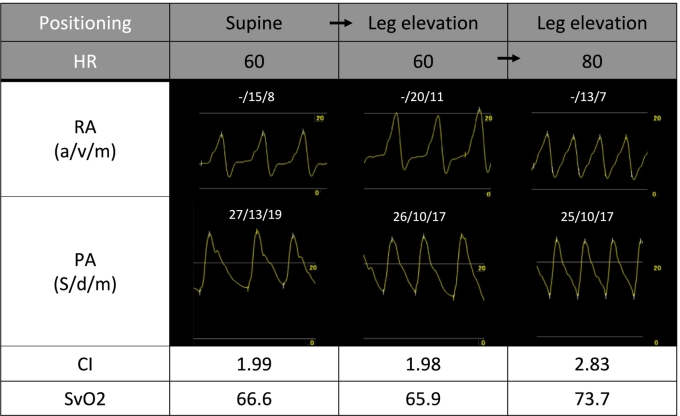
This figure contrasts the hemodynamic changes observed during right heart catheterization when the heart rate is set at 60 bpm versus 80 bpm. The increase in heart rate decreases the right atrial pressure and increases the cardiac output and mixed venous oxygen saturation, which underscores the treatment strategy's efficacy through heart rate optimization. HR, heart rate; RA, right atrium; PAP, pulmonary artery pressure; CI, cardiac index; SvO₂, mixed venous oxygen saturation.

Right heart catheterization at 60 bpm (Fig. 2) showed a rise in right atrial pressure from 8 to 11 mmHg during leg lifting, while pulmonary artery pressure, cardiac output, and SvO₂ remained unchanged. Increasing the pacing rate to 80 bpm improved hemodynamics, lowering right atrial pressure (from 11 to 7 mmHg). At 80 bpm, preload reserve appeared enhanced compared to 60 bpm, as shown by reduced right atrial pressure during leg lifting, increased cardiac index (from 1.98 to 2.83 mL/min/m^2^), and SvO₂ (from 65.9 % to 73.7 %). Consequently, the pacemaker was set to 80 bpm. Although the patient was on a beta-blocker, we did not reduce the dose due to a history of AF tachycardia.

**Fig. 3:**
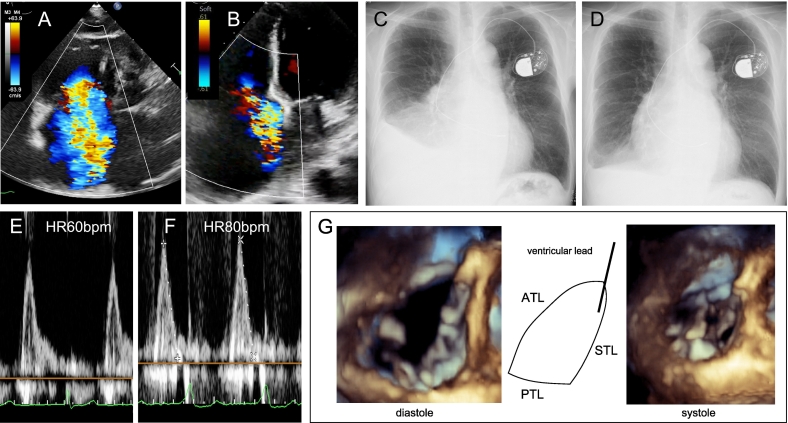
This figure demonstrates the longitudinal changes in tricuspid regurgitation and pleural effusion before and after heart rate optimization. (A) Massive tricuspid regurgitation before rate adjustment. (B) Moderate-to-severe tricuspid regurgitation after 5 months at 80 bpm. (C) Chest X-ray showing bilateral pleural effusion before rate adjustment. (D) Chest X-ray after 5 months showing resolution of pleural effusion. (E) Transmitral flow at baseline, showing E wave ending before QRS onset. (F) Transmitral flow after pacing at 80 bpm, also preserving E wave before QRS. (G) 3D transthoracic echocardiography confirming no interference of the pacemaker lead with the tricuspid valve. ATL, anterior tricuspid leaflet; STL, septal tricuspid leaflet; PTL, posterior tricuspid leaflet.

This rate adjustment reduced the incidence of hypotension. Echocardiographic comparison at baseline and 5 months showed improvement in both right and left ventricular function. Transmitral flow analysis before and after the rate adjustment showed that in both instances, the E wave terminated before the onset of the paced QRS complex, indicating that left ventricular filling was not adversely affected by the increased heart rate (Fig. 3E, F). Pleural effusion, assessed by chest X-ray and ultrasound, decreased in association with improved hemodynamics rather than dialysis schedule changes. After 5 months, pleural effusion was reduced (Fig. 3D), and TR severity decreased to moderate (Fig. 3B, Video 2), with a reduction in vena contracta width from 15.2 mm to 8.3 mm, as shown by grayscale and color Doppler imaging. The online videos demonstrate reduced regurgitant jet area and improved leaflet coaptation at the optimized heart rate, supporting continued conservative management. Following adjustment to RV pacing at 80 bpm, right and left ventricular function slightly declined—TAPSE decreased from 34 mm to 30 mm, RV-FAC from 49 % to 44 %, and left ventricular ejection fraction from 59 % to 52 %—but this did not result in any clinical deterioration.

Despite improvement, moderate TR persisted and may have contributed to ongoing symptoms such as intradialytic hypotension. Accordingly, continued follow-up is planned. Further optimization of pacemaker settings and consideration of advanced therapies will be evaluated if needed. The patient remained clinically stable with no worsening in echocardiographic findings or heart failure symptoms during the one-year follow-up.

## Discussion

This case highlights key clinical insights into determining the optimal heart rate in severe heart failure. Echocardiography and right heart catheterization help guide pacing rate adjustments to optimize cardiac output. Doppler analysis of atrioventricular valve inflow is a simple and useful echocardiographic technique. Increasing the pacing rate has emerged as a viable strategy to improve hemodynamics.

Several reports have assessed optimal heart rate using the mitral inflow Doppler waveform, based on E–A wave overlap in sinus rhythm [[1], [2], [3]]. However, this method is impractical in AF, where the A wave is absent. In AF, where the E wave dominates ventricular inflow, optimal heart rate can instead be inferred from the timing of atrioventricular valve closure following the E wave. The valve closure artifact within the Doppler waveform supports this evaluation. In our case, the optimal rate was identified by adjusting the pacemaker setting during echocardiography, using tricuspid valve inflow rather than mitral, due to predominant right heart failure from TR. Right heart catheterization confirmed 80 bpm as optimal, enabling avoidance of surgery.

Beta-blockers are commonly used for heart rate control in AF. Patients with persistent AF and a history of chronic β-blocker use often exhibit relative bradycardia. In such cases, reducing or discontinuing β-blockers may help achieve optimal heart rate. This is especially relevant in heart failure with preserved ejection fraction, where β-blockers lack prognostic benefit, underscoring the importance of dose adjustment based on optimal rate assessment.

The optimal heart rate in heart failure remains debated, especially following the introduction of ivabradine, which targets tachycardia reduction [4]. Although relative bradycardia without symptoms (e.g. fainting or unsteadiness) may not meet criteria for pacemaker implantation, increasing heart rate to improve hemodynamics should be considered. We previously reported a case where evaluating the optimal heart rate using temporary pacing during right heart catheterization in severe left heart failure with relative bradycardia stabilized the patient post-pacemaker implantation [5]. That case showed that increasing heart rate to shorten diastolic period—without E and A wave fusion—could enhance cardiac output without reducing stroke volume.

In this case, regulating the heart rate to increase cardiac output may have induced reverse remodeling of the right heart, reducing TR via a decrease in tricuspid annular area. Indeed, the tricuspid annular diameter decreased from 59 to 49 mm in the RV-focused apical four-chamber view.

Evaluating lead-induced TR is essential when severe TR develops after pacemaker implantation [6]. A 3D transthoracic echocardiogram is required to determine whether the RV lead crosses the tricuspid valve apparatus. If lead-induced TR is diagnosed, careful assessment is warranted, as TR may improve after lead removal. In this case, the RV lead did not traverse the tricuspid apparatus, ruling out lead-induced TR.

The long-term benefits of atrial pacing at 80 bpm remain unclear. Some trials have shown that lower resting sinus rates offer protection in stable heart failure. Subgroup analysis of the SHIFT trial showed lower risk of cardiovascular death or heart failure hospitalization with slower resting heart rates [7]. Likewise, the BEAUTIFUL trial showed higher cardiovascular risk in patients with heart rates ≥70 bpm [8]. Nonetheless, this case illustrates the feasibility of re-evaluating optimal pacing rate during the chronic phase via echocardiography-guided pacemaker adjustment. A 5-month follow-up confirmed the sustained effectiveness of 80 bpm.

In conclusion, this case highlights the importance of tailored heart rate management in severe heart failure using pacemaker adjustment and Doppler analysis. It underscores the need for personalized strategies to improve patient outcomes.

The following are the supplementary data related to this article.

Video 1 Color Doppler echocardiography showing tricuspid regurgitation before rate adjustment (corresponding to Fig. 3A). Video 1

Video 2 Color Doppler echocardiography showing tricuspid regurgitation after rate adjustment (corresponding to Fig. 3B). Video 2

## Consent statement

Written informed consent was obtained from the patient for the publication of this case report, including accompanying images.

## Declaration of competing interest

The authors declare that they have no conflicts of interest.
